# Key findings from a prospective trauma registry at a regional hospital in Southwest Cameroon

**DOI:** 10.1371/journal.pone.0180784

**Published:** 2017-07-19

**Authors:** Alain Chichom-Mefire, Obieze C. Nwanna-Nzewunwa, Vincent Verla Siysi, Isabelle Feldhaus, Rochelle Dicker, Catherine Juillard

**Affiliations:** 1 Department of Surgery, Faculty of Health Sciences, University of Buea and Regional Hospital Limbe, Buea, Cameroon; 2 Center for Global Surgical Studies, Department of Surgery, University of California, San Francisco, San Francisco, California, United States of America; University of Kansas Medical Center, UNITED STATES

## Abstract

**Introduction:**

Trauma is a leading cause of morbidity and mortality worldwide. Data characterizing the burden of trauma in Cameroon is limited. Regular, prospective injury surveillance can address the shortcomings of existing hospital administrative logs and medical records. This study aims to characterize trauma as seen at the emergency department (ED) of Limbe Regional Hospital (LRH) and assess the completeness of data obtained by a trauma registry.

**Methods and findings:**

From January 2008 to October 2013, we prospectively captured data on injured patients using a strategically designed, context-relevant trauma registry instrument. Indicators around patient demographics, injury characteristics, delays in accessing care, and treatment outcomes were recorded. Descriptive, bivariate, and multivariate statistical analyses were conducted.

About 5,617 patients, aged from 0.5-95years (median age of 26 years), visited the LRH ED with an injury; 67% were male. Students (27%) were the most affected occupation category. Road traffic injuries (RTIs) (56%), assault (22%), and domestic injuries (13%) were the leading causes of injury. Two-thirds of RTIs were motorcycle-related. Working in transportation (AOR 4.42, p<0.001) and law enforcement (AOR 1.73, p = 0.004) were significant predictors of having a RTI. The trauma registry showed a significant improvement in completeness of all data (p<0.001) and it improved over time compared with previous administrative records. However, proportions of missing data still ranged from 0.5% to 8.2% and involved respiratory rate or Glasgow Coma scale.

**Conclusions:**

Implementation of a context-appropriate trauma registry in resource-constrained settings is feasible. Providing valuable, high-quality data, the trauma registry can inform trauma care quality improvement efforts and policy development. Study findings indicate the need for injury prevention interventions and policies that will prioritize high-risks groups, such as those aged 20–29 years, and those in occupations requiring frequent road travel. The high incidence of motorcycle-related injuries is concerning and calls for a proactive solution.

## Introduction

Trauma, a leading cause of morbidity and mortality worldwide, accounts for about 10% of the global burden of disease and over five million deaths annually [1][2]. Low- and Middle-Income Countries (LMICs) are disproportionately affected by trauma, with about 90% of injury-related mortality occurring in these settings [3]. The incidence and burden of trauma in LMICs is growing due to poor road infrastructure, weak road safety policies and other factors [4,5]. In Cameroon, the burden of road traffic injuries (RTIs) continues to increase, rising from the 11^th^ to the 8^th^ leading cause of disability-adjusted life years (DALYs) between 1990 and 2010 [6].

Despite the growing trauma burden, there is inadequate data to characterize trauma and this hampers the understanding and improvement of trauma care in Cameroon [7–9]. Primary data that captures the epidemiology of trauma in LMICs is exceedingly scarce [3,10–12]. In Cameroon, administrative records are often the only existing source of trauma data but they bear considerable limitations [7]. Although reviews of hospital administrative and medical records can be a useful first step, these are often incomplete sources of information essential to the care of the acutely injured [7,13,14]. Administrative data are often inaccurate and may result in underreporting, missing data, and they often do not incorporate information pertaining to costs, outcomes, and key risk factors [15,16]. These limitations are typical of retrospective analyses of administrative records in Cameroon and similar settings [9,13,17–19].

To adequately respond to this growing trauma burden, the pattern and determinants of trauma need to be well characterized [20]. Routine prospective trauma surveillance can address the shortcomings of existing hospital administrative logs and medical records [14]. Trauma registries, which are vital tools for monitoring and enhancing trauma care, need to be developed and implemented in LMICs [21–24]. Data elicited from a strategically designed prospective registry can help determine health priorities, influence policies for emergency care, and guide appropriate resource allocation[25]. This study aims to assess the role and capacity of a prospective trauma registry in profiling the demographics, nature of injuries, clinical patterns, and health outcomes of patients arriving to Limbe Regional Hospital in Cameroon for trauma care.

We hypothesized that a locally developed trauma registry would provide information that characterizes the burden of trauma better than the previous administrative system of data collection [7] and could inform improvement of trauma care and related policy. To test this hypothesis, we assessed the demographic, injury characteristics, clinical patterns, and health outcomes data of trauma patients, as captured by the trauma registry at Limbe Regional Hospital, and we compared this data with other data sources in order to evaluate data quality.

## Methods

### Study setting

This study was conducted at Limbe Regional Hospital, an institution of 200 beds with an emergency and casualty department, outpatient department, and a general surgery unit with a 46-bed capacity [26]. Regional hospitals represent the third level of care on five-point scale of the hospitals in the Cameroonian health system, below which are the district hospitals and health centers. Limbe Regional Hospital serves as the principal referral hospital for a population of about 1.3 million people in the Southwest Region of Cameroon [27]. The Emergency Department (ED), available 24 hours a day, at Limbe Regional Hospital sees an estimated 140–470 trauma patients monthly [7,8].

### Study materials and data collection

A trauma registry (S1 Table) was created to replace the existing general patient registration protocol. The registry was designed to collect data on demographics, nature of the injury, delay in accessing care, clinical documentation [i.e., assessment and documentation of vital signs, Glasgow Coma Scale (GCS), Injury Severity Score (ISS), and Revised Trauma Score (RTS)], and outcome of care. Using a paper version of the registry (S1 Appendix), a research assistant filled out the demographic and non-clinical information, while clinicians fill out the clinical information of trauma patients into the registry. The research assistant then transferred this data to a Microsoft Excel spreadsheet [28]. Prior to the study, local providers were trained on how to use the instrument. From January 2008 to October 2013, 12 nurses, six general practice physicians, one surgeon, and a research assistant, all of whom worked in the ED, implemented the trauma registry, by gathering the data while running their daily shifts. Before inclusion in the study, each patient provided written informed consent. For unconscious patients, provisional administrative authorization was obtained and if the later the patient refused to participate, he was excluded from the study.

### Data analysis

Descriptive statistics were generated on the trauma registry, resulting in a profile of trauma patients based on: demographics, length of prehospital delay, clinical characteristics, type and mechanism of injury, and treatment outcome. Chi-square tests were applied to identify associations between demographic characteristics and nature of injury. Univariate and multivariate logistic regression models were developed to characterize identified associations between demographic characteristics and the nature of injury, particularly RTIs. Similar analyses examining treatment outcomes were deemed inappropriate given the limitations of data available. Data were analyzed using Microsoft Excel 2011 and Stata 12.0 [28,29]

### Ethical approval

This study received ethical approval from 1) the University of California, San Francisco Office of Ethics and Compliance, Human Research Protection Program & IRB, and 2) the University of Buea Ethical Committee and Institutional Review Board.

## Results

### Demographic characteristics

**Fig 1:**
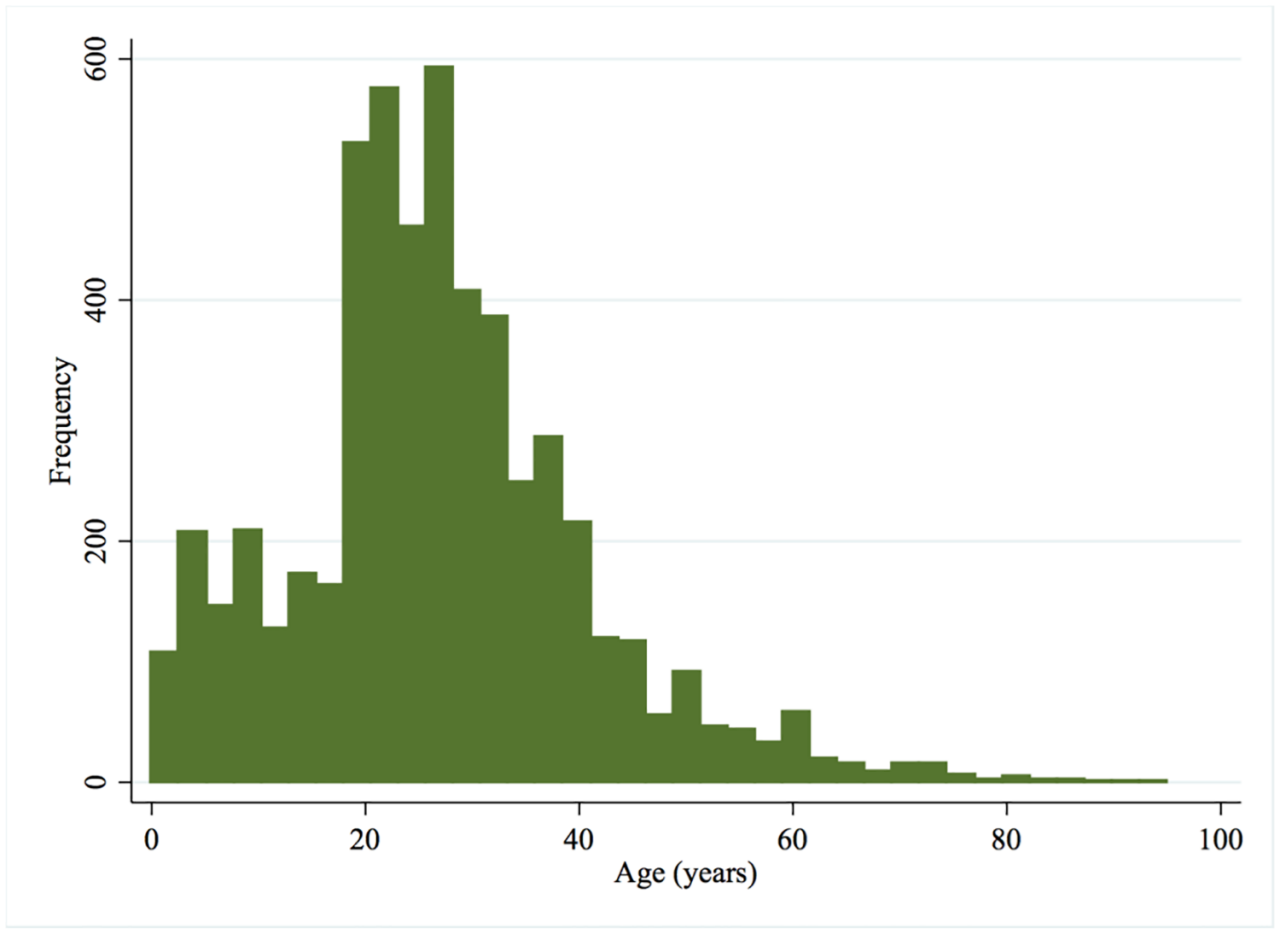
Age distribution of trauma patients (n = 5,503).

**Fig 2:**
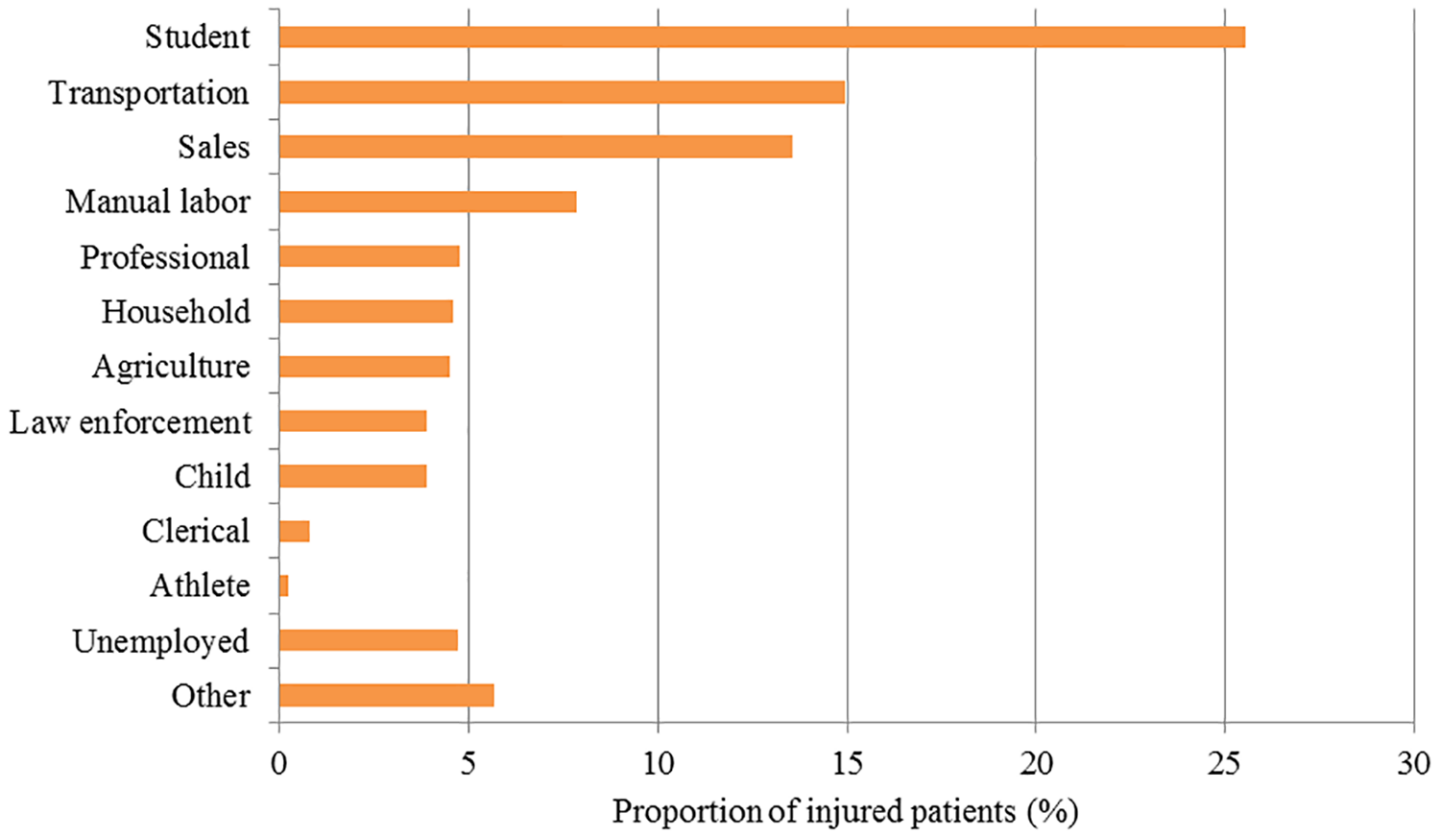
Occupation of injured patients (n = 5,337).

A total of 5,617 patients, were seen at Limbe Regional Hospital ED over the study period. Two-thirds of the patients were male (67.6%) (Table 1). The mean age was 26.8 years [CI: 26.4, 27.2] and patients ranged from 0.5 to 95 years old, with majority (36.5%) being 20 to 29 years old (Fig 1). Most patients (88.2%) were residents of Limbe municipality (Table 1). Students comprised the highest proportion of injured patients (25.5%), followed by those working in transportation (15.0%), and sales (13.6%) (Fig 2).

**Table 1:** Profile of injured patients arriving to ED of Limbe Regional Hospital, Jan 2008 to Oct 2013 (N = 5,617).

Characteristic	n	%
Sex		
Male	3,797	67.6
Female	1,781	31.7
Missing	39	0.7
Age		
0–9 years	598	10.7
10–19 years	841	15.0
20–29 years	2009	35.8
30–39 years	1234	22.0
40–49 years	468	8.3
50–59 years	198	3.5
60+ years	155	2.8
Missing	114	2.0
Municipality of residence		
Limbe	4,952	88.2
Outside Limbe	539	9.6
Missing	126	2.2
Mechanism of injury		
Road traffic injury (RTI)	3,097	55.1
Assault	1,223	21.8
Domestic accident	732	13.0
Fall	233	4.2
Labor accident	81	1.4
Other	210	3.7
Missing	41	0.7
Anatomical location of injury		
Head	1,326	23.6
Cervical spine	112	2.0
Face and neck	1,301	23.2
Chest	289	5.2
Abdomen	130	2.3
Dorsal or lumbar spine	129	2.3
Upper limb	1,655	29.5
Lower limb	2,170	38.6
Other	37	0.7
Missing	238	4.2
Injury Severity Score (ISS)		
Mild (<9)	4,829	86.0
Moderate (9–15)	254	4.5
Severe (16–24)	14	0.3
Profound (> = 25)	60	1.7
Missing	460	8.2
Pre-hospital delay		
Less than 1 hour	3,763	67.0
1 to 6 hours	1,219	21.7
More than 6 hours	174	3.1
Missing	461	8.2
Outcome		
Formally Discharged	5,022	89.4
Ward Admission	357	6.4
Referral/ Transfer	129	2.3
Death	22	0.4
Discharged Against Medical Advice	7	0.1
Missing	80	1.4

### Nature of injury

RTIs (55.5%) were the leading cause of injury followed by assault (21.9%) (Table 1). Of the patients who sustained RTIs, 53.9% were passengers, 28.3% drivers and 17.8% were pedestrians. Among patients that suffered an RTI, 34.7% involved a motorcycle alone, 22.7% involved a car against a motorcycle, 14.8% car alone, 11.6% motorcycle against pedestrian, and 7.0% car against pedestrian.

Analysis of mechanism of injury and sex revealed statistically significant differences in how men were injured compared to women (p<0.001). Men were significantly more likely to sustain an RTI (p = 0.002), while women were more likely to be injured in a domestic injury (p<0.001). Data also indicated statistically significant differences in mechanism of injury by age group. Children under 19 years of age were not as likely as other age groups to sustain an RTI (p<0.001). However, individuals 20 to 29 years old were more likely to be injured in a road traffic incident compared to all other age groups (OR 1.24, p<0.001). Among men sustaining RTIs, 40.3% were between the ages of 20 and 29 years, while 24.4% were between 30 and 39 years old. Similarly, among women with RTIs, 32.9% were between the ages of 20 and 29 years; however, 20.2% were between 10 and 19 years old.

Among patients sustaining RTIs, 25.0% were students, 22.6% worked in transportation, and 14.0% in sales. RTIs were the leading cause of injury across all occupational groups, most notably making up 79.4% of injuries of those working in transportation. Injured patients of in this occupational group were more likely to sustain an RTI (OR 3.72, p<0.001). Children were most often injured in domestic injuries (37.5%) followed closely by road traffic incidents (35.7%). There were also statistically significant differences between municipality of residence and mechanism of injury (p<0.001). In particular, injured persons living outside of the Limbe municipality were more likely to arrive to the emergency department with an RTI (OR 2.16, p<0.001). Multivariate analysis adjusting for patient sex, age, occupation, and municipality of residence indicated that working in transportation (AOR 4.42, p<0.001), law enforcement (AOR 1.73, p = 0.004), and living outside of the Limbe municipality (AOR 2.06, p<0.001) were significant predictors of having an RTI.

### Clinical characteristics

Most patients were injured in only one (69.7%) or two (21.4%) anatomical locations. The remaining 4.7% of patients were injured in three or more locations. The most common locations of injury were extremities (68.1%) (Table 1). ISS was determined for 5,157 patients (91.8%) with the overwhelming majority of injuries being mild in severity (ISS<9) (93.6%) (Table 1). GCS was recorded for 3,707 (66.0%) of injured patients, of whom, 97.8% were mildly injured. RTS was recorded for 467 patients (8.3%). Twenty-two deaths occurred. Eighteen of the deceased had a documented GCS, of which 17 were classified as severely injured. About 13 of the deceased were awarded an ISS, of which only four were classified as severe injuries while the remaining nine were considered to have mild injuries.

### Completeness of data

**Fig 3:**
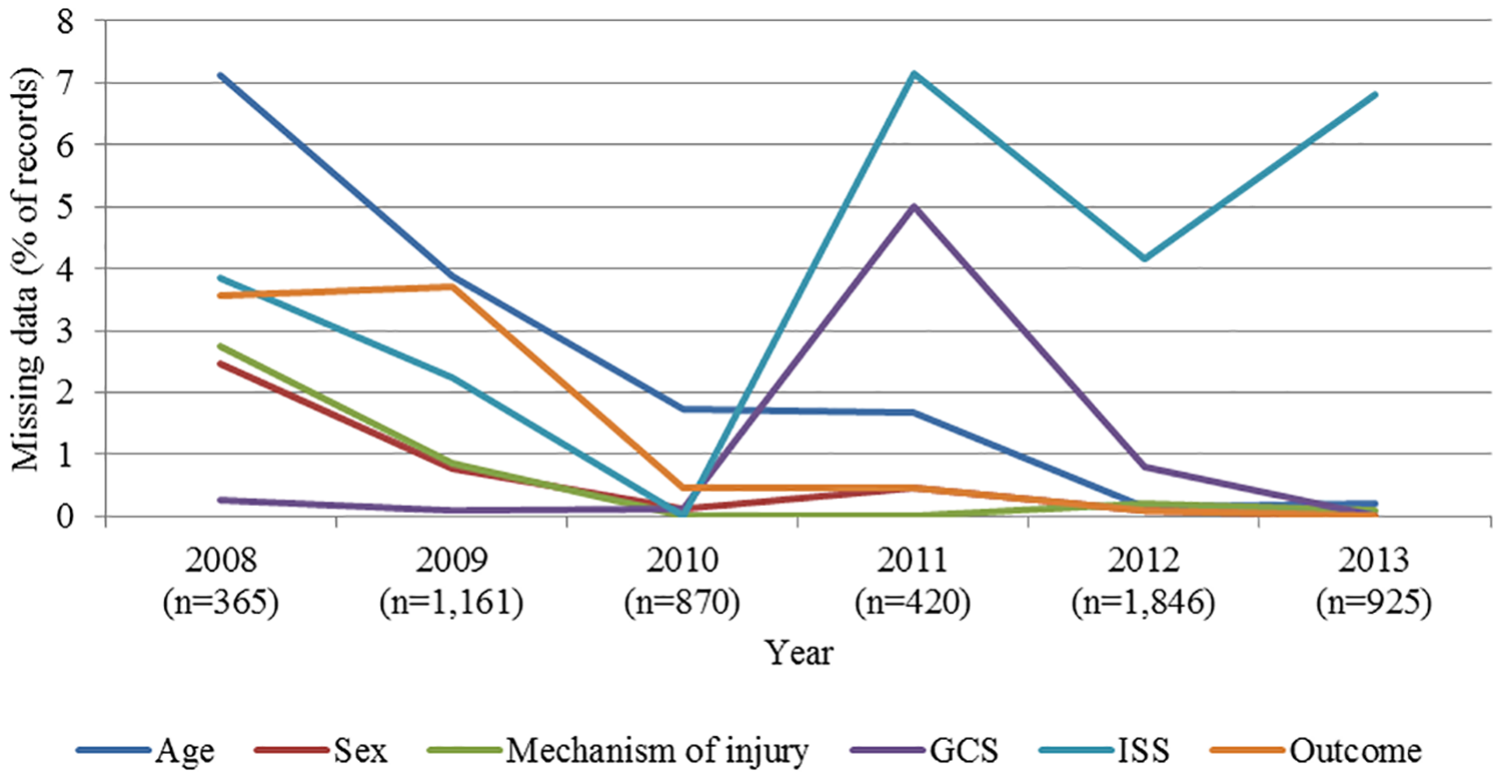
Proportion of missing data in trauma registry by indicator, 2008–2013 (N = 5,617).

The Limbe trauma registry collected significantly more data for all variables compared to the retrospective administrative hospital logs at the same facility (Table 2). A similar pattern was observed when comparing the Central Hospital Yaounde trauma registry with their administrative data [7,8]. However, the number of records collected annually in the Limbe trauma registry was inconsistent. The proportion of missing data for most variables typically ranged from 0.5–14.9%, except for RTS, which was not documented in 57.3% of cases. The quality of data collection using the trauma registry generally improved over time and the lowest rate of missing data was seen in years 2010 and 2013 (Fig 3). Clinical and calculated variables, such as GCS, ISS, and RTS, were consistently difficult to collect across the years. The varying proportions of missing data for blood pressure and respiratory rate were parallel across years of data collection, both peaking at 30.5% missing in 2011.

**Table 2:** Comparison of the Limbe Regional Hospital trauma registry trauma-related indicators with other institutional data sources for data completeness.

			Proportion complete (%)							
Hospital name	Source of data		Occupation	Anatomical location	Blood pressure	Respiratory rate	Glasgow coma score	Injury severity score	Patient transport time	Outcome of Emergency Department treatment
Limbe Regional Hospital data completeness	Trauma registry (n = 5617) (60 months)		95%	96%	71%	26%	92%	66%	92%	99%
	Administrative records (n = 1713) (12 months) [10]		79%	83%	13%	2%	8%	7%	4%	45%
		p-value	<0.001	<0.001	<0.001	<0.001	<0.001	<0.001	<0.001	<0.001
Central Hospital Yaounde data completeness	Pilot Trauma registry (n = 2855) (6 months) [15]		93%	96%	98%	99%	97%	96%	92%	94%
		p-value	<0.001	1.00	<0.001	<0.001	<0.001	<0.001	1.00	<0.001
	Administrative records (n = 6,324) (12 months) [17]		-	98%	-	-	-	-	-	99%
		p-value	-	<0.001	-	-	-	-	-	1

## Discussion

Limbe Regional Hospital encounters a high trauma volume, which is chiefly due to traffic injuries and assault. Patients were predominantly young (10 to 39 years old), male, and residents of Limbe municipality presenting with mild injuries at one or two anatomical locations. Most patients arrived at the emergency department within an hour of being injured. These visits most often led to formal discharge.

Study findings support existing literature showing that young males are more likely to be victims of trauma, a phenomenon that can cause significant social and economic hardship to families [8,30–33]. Such hardship could be a result of expensive out-of-pocket health care costs or the loss of household earnings incurred during the period of treatment, subsequent disability, or both [34]. From this perspective, trauma poses a considerable economic risk to entire households, warranting further research and investment focused on increasing access to adequate trauma care as well as effective injury prevention. If universal health coverage is to be achieved, implementing sustainable schemes for financial risk protection of individuals that are at an increased risk for trauma must be a priority.

Two-thirds of all injuries presenting to the hospital were RTIs. This is similar to findings in other sub-Saharan African countries, where RTI account for approximately 40–90% of all injuries [17,35]. Unsafe transportation options, poor road infrastructure, or ineffective and/or poorly enforced policies to ensure road safety may contribute to the high burden of RTIs in the region [36]. The occupations most often injured were students (25.5%) followed by transportation workers (15.0%) and salespersons (13.6%). With these groups traveling regularly, this finding is presumably tied to RTIs as the leading cause of injury. Public education efforts to improve awareness and road safety should target these high-risk populations.

About 67% of RTIs (37% of all injuries) were motorcycle-related, making motorcycle-associated injury the leading mechanism of injuries in this setting. Studies in other settings indicate similar rates of motorcycle-associated injuries [37–39]. Considering that motorcycles account for over 30% of public transportation in Cameroonian cities, there may be a need to explore safer transportation alternatives [40]. Municipal governments in Nigeria and Liberia, for instance, have banned the use of motorcycles for public transportation and have seen a subsequent reduction in RTI incidence [32,41,42]. Indonesia requires that public transportation vehicles have at least three wheels [43]. Ancillary measures may include proper road construction to enhance road safety for motorists and also pedestrians.

Assault (21.7%) and domestic injuries (13.0%) were the second and third leading mechanism of injury, respectively. The high rate of interpersonal violence is an interesting finding in the absence of civil unrest or documented communal clashes. This registry does not capture the exact mechanism of injuries. Efforts to further explore the nature of these mechanisms of injury could inform interventions aimed at prevention or addressing underlying causality.

The implementation of the trauma registry in Limbe led to improvement in the data collection and quality. The amount and quality of data collected via the Limbe trauma registry suggest that adequate implementation requires continued commitment and oversight. Despite some fluctuations in the amount and quality of data collected, there was general improvement in the completeness of data collected for indicators over the course of the study period. This may suggest that data collection using the trauma registry became routine over time, contributing to more regular recordkeeping of specified indicators. Challenges persisted in the collection of calculated clinical indicators, such as GCS, ISS, and RTS. However, given the logistical and technological requirements of various trauma-scoring systems, this may speak to the broader challenge of accurately applying these scores in resource-limited settings.

Local stakeholder buy-in, adequate and motivated workforce, and secure funding are crucial elements that support the sustainability of a trauma registry. While some studies suggest that registries must be locally-driven to be sustainable, the role of international or external partners in funding, developing, implementing, and evaluating trauma registries is also key [44]. Electronic registries may not be feasible in every setting, but where the capacity for electronic registries exists, simple platforms or software that are user-friendly and familiar to the local workforce, are advantageous.

A trauma registry can serve as tool for developing and monitoring quality improvement interventions that involve reliable clinical documentation. In comparison with secondary data obtained from administrative records, the use of the trauma registry for data collection was associated with a higher level of data completeness for all variables, particularly blood pressure, respiratory rate, GCS, ISS, and patient transport time [7,9]. This hints that trauma registries are not only feasible but can also improve the quality of clinical documentation in LMIC settings. The registry can also serve as a basis for the development of an electronic medical records system appropriate for this setting.

A limitation of this hospital-based study is that the data collection approach limits the sample population studied to injured persons seeking formal care at the facility. Thus, it is possible that the incidence and epidemiology of injury is different when examined at the community level. While the proportion of missing data is typically low for indicators included in the Limbe Regional Hospital trauma registry, the variation in annual number of records highlights the possibility of inconsistencies in data collection or overall implementation of the registry. In terms of data collected, the failure of severity scores to correlate with patient outcomes suggests broader challenges of applying these scoring systems in this setting. Imaging equipment or infrastructures (e.g. CT machine, electricity) are required to accurately estimate the ISS. Often these may be unavailable intermittently or altogether. Such limitations may affect the accurately and continuously estimate and report ISS, and may thus have contributed to the low documentation rate of ISS and the inability of the estimated ISS to accurately predict mortality. Furthermore, resource limitation may also contribute increased likelihood of mortality despite an apparently mild or moderate injury severity score. Though beyond the scope of this particular study, it is important to explore context-appropriate injury severity scoring systems that may enhance the utility of the trauma registry in clinical care provision. Physiologic injury scoring systems, or those that do not rely on the availability of sophisticated equipment, may be more feasible in resource-limited settings. This may be supported by the fact that over time, there was less missing data for GCS compared with the ISS (Fig 3).

## Conclusions

This study shows that the implementation of a locally-developed trauma registry is feasible and sustainable, providing valuable, higher quality data to inform our understanding of injury as well as trauma care and systems in this setting. The high prevalence of motorcycle-related injuries raises concerns and the need for a proactive approach towards reducing the incidence of these injuries. Improving road safety and infrastructure, provision of safer public transportation systems, and the formulation and implementation of policies to ensure road and passenger safety will be critical to reducing the risk and occurrence of trauma in the region.

The availability of trauma registry data in LMICs will enhance the understanding of the local and global surgical disease burden. Such understanding will catalyze the prioritization of surgical care delivery in LMICs and facilitate the development and implementation of targeted capacity building and quality improvement interventions that are context-appropriate.

## Supporting information

S1 Table The original data set. (XLS)

S1 Appendix The data collection tool used in our study. (DOC)
