# Supplementary material for: Key findings from a prospective trauma registry at a regional hospital in Southwest Cameroon
Source: PLoS One. 2017 Jul 19;12(7):e0180784. doi: 10.1371/journal.pone.0180784 (PMC5516986; doi:10.1371/journal.pone.0180784)
Supplement: S1 Appendix — (DOC) [file pone.0180784.s002.doc]

**PROSPECTIVE TRIAL ON INJURIES**

**Data collection sheet**

*Please, kindly fill this form after obtaining written concern from the patient or his relatives; for help, please call 77 53 05 32.*

**Patient identification: Date __________________ Sheet No: ________**

Name (optional): ____________________________________ Age: __________ Sex______

Profession_______________________________ place of residence_____________________

**Type of trauma (choose one)**

Road traffic accident_______ Domestic accident________ fall ______________ assault__________

Burns _________ labour accident__________ other (specify) ____________________________

**Type of accident (choose one; to be filled only for road traffic accidents):**

Car alone_______ Car against car________ Car against motorcycle_______ Motorcycle alone______

Car against pedestrian______ motorcycle against pedestrian____ other_____ (indicate) _________________

**Position of patient:** Driver __________ passenger ___________ pedestrian____________

**Delay before arrival to hospital (choose one):**

Less than 1 hour___________ 1 to 6 hours ____________ more than 6 hours ___________

**Location of injury (choose one or more):**

Head _________ cervical spine _________ face and ENT _________ chest __________ abdomen __________

Dorsal or lumbar spine ___________ upper limb ___________ lower limb ____________

**Glasgow coma scale: ___________/15. Respiratory rate ____________ systolic blood pressure __________**

**Please, describe the lesions (as detailed as possible):**

**Outcome of patient after casualty:**

Discharged ________ admission in ward _________ Transfer or referral ________ death __________

***Please, box below to be filled by doctor***

**ISS** (injury severity score): ______________________________

**Revised trauma score:**

- respiratory rate ___________/4
- Systolic blood pressure _______________/4
- Glasgow coma scale: _____________/4
- Total: __________________/12

***For patients admitted in the ward:***

**Duration of admission** _______________________

**Outcome:**

Discharge ___________ death ________________ transfer or referral _______________
